# High-throughput sequencing study of the effect of transabdominal hysterectomy on intestinal flora in patients with uterine fibroids

**DOI:** 10.1186/s12866-020-01779-7

**Published:** 2020-04-15

**Authors:** Wantong Wang, Yibing Li, Qijun Wu, Xin Pan, Xinhui He, Xiaoxin Ma

**Affiliations:** Department of Obstetrics and Gynecology, Shengjing Hospital of China Medical University, Shenyang, 110004 China

**Keywords:** Intestinal flora, Uterine fibroids, Hysterectomy, Estrogen, High-throughput sequencing, 16sRNA

## Abstract

**Background:**

To investigate the effect of transabdominal hysterectomy on the diversity of the intestinal flora in patients with uterine fibroids. Patients with uterine fibroids were selected from September 2018 to December 2018, in the Department of Obstetrics and Gynecology, Shengjing Hospital of China Medical University, and stool specimens were collected from patients before and after surgery.

**Results:**

High-throughput sequencing of the 16S rRNA gene was used to detect the changes in microbial community structure and diversity, and the effects of total hysterectomy on the intestinal flora were further analyzed. Estrogen levels decreased after trans-abdominal hysterectomy. High-throughput sequencing showed that after abdominal hysterectomy, the abundance and diversity of the intestinal flora decreased. The abundance changes were mainly due to *Proteobacteria*, where their abundance increased.

**Conclusions:**

Trans-abdominal hysterectomy changes the intestinal flora of the body by lowering the level of estrogen in the body, which reduces the diversity and abundance of the intestinal flora.

## Background

Uterine fibroids are common benign tumors that manifest in 30–50 year-old women. According to autopsy statistics, approximately 20% of women over the age of 30 have uterine fibroids. The growth and persistence of uterine fibroids depends on the woman’s estrogen and progesterone levels, and the synthesis of estrogen is affected by many factors in vitro and in vivo [1]. The change in estrogen levels is an independent contributor to the onset of uterine fibroids in women. The synthesis and secretion of estrogen are affected by vitamin D and E, and trace elements, such as iodine and selenium. Women with uterine fibroids and trace elements in tumors have statistically significant differences, when compared to normal women, with zinc and copper as the main features [2, 3]. Related studies have also confirmed that in addition to the effects of estrogen and progesterone on uterine fibroids, trace elements, growth factors and immune cells in the body are also closely correlated to uterine fibroids. The TNF-α (tumor necrosis factor-α) produced by phagocytic cells can cause proliferative changes in injured smooth muscle cells. TNF-α is correlated to the occurrence of uterine fibroids, which may be due to the suppression of the immune state of the body [4]. Growth factors in the body are correlated to the occurrence of uterine fibroids. The most closely correlated are EGF (epidermal growth factor), VGEF (vascular endothelial growth factor), and TGF (transforming growth factor). Patients with uterine fibroids have higher receptors for related growth factors, when compared to normal uterine myometrium. Growth factors are affected by estrogen, and increase the proliferation and division of uterine fibroid cells [5–7].

For women with uterine fibroids without fertility requirements, total hysterectomy is one of the more classic procedures. Total hysterectomy has an effect on the patient’s sex hormone levels. Most women who undergo total hysterectomy would exhibit varying degrees of sexual function reduction [8]. A study revealed that the effect of sex hormone levels on patients with different ranges of hysterectomy was not statistically significant. However, after the total hysterectomy, the sex hormone levels were significantly lower than those before surgery [9].

A large number of gut microbes constitute the most complex micro-ecological system in the human body [10]. The changes in the composition of the human intestinal flora affect the body’s movement, immunity, and endocrine and sex hormone levels [11–13]. The large number of human intestinal microbes affect the physiological functions of the human body at all times, and has profound effects on the synthesis and secretion of hormones, trace elements, growth factors and immune system. The intestinal flora and host have a mutually beneficial relationship, and are interdependent. The intestinal flora plays an important role in regulating metabolic pathways, synthesizing essential components such as vitamins, and promoting the establishment of the immune system. The flora provides nutrients and a suitable living environment, and the stable balance between the host and intestinal flora plays an indispensable role in maintaining the health of the human body [14]. The intestinal flora plays a vital role in regulating several chronic diseases, including obesity, cardiovascular disease and kidney diseases [15–19]. The balance in intestinal flora composition is the key to maintaining intestinal function and systemic homeostasis [20]. A study conducted in 2018 revealed that estrogen could inhibit the overgrowth of *Bacteroides fragilis*,*Escherichia coli* and *Fusobacterium nucleatum* to maintain the homeostasis of intestine mucosa [21]. Estrogen deficiency can lead to a marked reduction in intestinal flora biodiversity and the number of beneficial bacteria with immune regulation, while the number of conditional pathogens is elevated, thereby triggering a series of inflammatory immune responses that lead to a disease. In recent years, some high-throughput sequencing and other cutting-edge technologies have promoted the study of the intestinal flora. However,there remains little knowledge about uterine fibroids and intestinal flora. The present study analyzed the differences in E2 (estradiol), AMH (anti-Mullerian hormone) and FSH (follicle-stimulating hormone) (FSH) before and after transabdominal hysterectomy, and further analyzed the impact on the diversity of the human intestinal flora.

## Results

### General clinical data

We collected fresh fecal samples from 15 patients, a total of 30 samples, 15 of which were preoperative specimens and 15 postoperative specimens. We compared E2, AMH, and FSH in the RS1 group and RS2 group. The AMH level of the RS1 group was lower than that of the RS2 group, but the difference was not statistically significant. The FSH level was higher in the RS1 group than the RS2 group, but the difference was not statistically significant. The E2 level was significant higher in the RS1 group. Therefore, we believe that ovarian function was greater in the RS1 group compared to the RS2 group (Table 1).

**Table 1:** Comparison of ovarian function between the two groups

	Group		T value	*P* value
	RS1(*n* = 15)	RS2(n = 15)		
AMH (nmmol/L)	4.78 ± 1.75	4.01 ± 1.00	1.82	0.09
E_2_(pmol/L)	460.71 ± 303.08	229.36 ± 110.19	2.65	0.02
FSH (IU/L)	11.86 ± 11.77	19.47 ± 28.29	−0.94	0.36

### Study of the structure of intestinal flora in patients with undergoing transabdominal hysterectomy

**Fig. 1:**
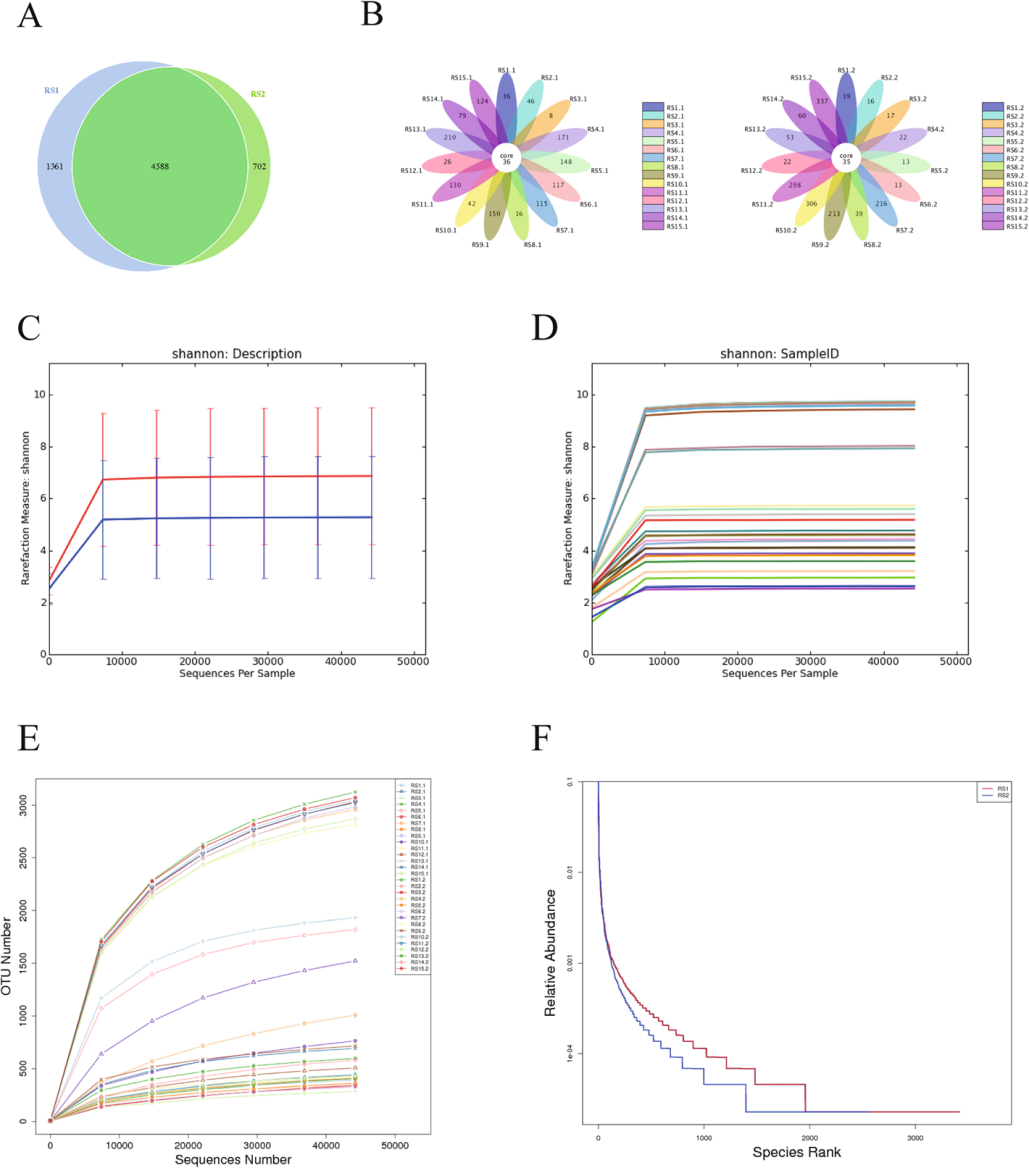
Comparison of the the structure of intestinal flora between patients with uterine fibroids before undergoing transabdominal hysterectomy (group RS1) and patients with uterine fibroids after undergoing transabdominal hysterectomy (group RS2).**a** Veen diagrams of OTUs .**b** Petal plot: Each petal in the petal diagram represents a sample, different colors represent different samples, the core number in the middle represents the number of OTUs common to all samples, and the number on the petal represents the sample unique OTU number.**c** Shannon description in the two groups .**d** Shannon description among each sample.**e** Rarefaction Curve.f Rank Abundance

A total of 2,083,203 sequences were obtained from 30 samples from the 2 groups, with an average of 73,170 sequences per sample. After quality control, 69,440 valid data were obtained, and the quality control efficiency was 94.96%. The mean number of sequences in the RS1 group was 70,122.73 ± 54.71, and the mean number of sequences in the RS2 group was 68,755.47 ± 5159.97, where the difference between the two groups was not statistically significant (*P* = 0.314). The sequence was clustered into OTUs (operational taxonomic units) with 97% identity. A total of 6651 OTUs were obtained, with an average of 221 OTUs per sample. Among them, the RS1 group had 5949 OTUs and the RS2 group had 5290 OTUs. Comparing the number of unique and common OTUs in the two groups according to the Veen chart, the OTU number composition and similar situation of the sample could be compared. The number of OTUs shared by the RS1 and RS2 groups was 4588, and the number of unique OTUs was 1361 in the RS1 group and 702 in the RS2 group. The Veen diagram showed that the diversity of RS1 was significantly higher than that of RS2. The petal plot indicated the number of OTUs contained in each sample of the RS1 and RS2 groups (Fig. 1A, B).

In this study, the statistical analysis of the sample at 97% similarity level produced a rarefaction curve, and a significant plateau appeared on the starting curve of 7737 sequences, indicating that the sequencing depth was close to saturation, increasing the sequencing depth at 97% similarity. No more bacterial species could be found at the top. The combination of the rarefaction curve and the Shannon diversity curve indicated that the amount of data in this study was reasonable, the sequencing depth was sufficient; the detection rate of the bacterial species of the sample was close to saturation, meeting the requirements of subsequent bioinformatics analysis (Fig. 1C, D, E). Second, by analyzing Good’s coverage index, the sequencing coverage of each sample was over 98%. 16S rRNA gene sequencing was effective in this study and represented more than 98% of the bacterial species in each sample, and the coverage of the bacterial species was good. The rank-abundance curve was steep, indicating that sample distribution was uneven, and there could have been a dominant flora; the curve span was large, indicating that the abundance of the species was high. As shown in (Fig. 1F), the RS1 curve had a wide and flat span on the horizontal axis, indicating that species richness and uniformity of the RS1 group were better.

The analytical indices included ACE, Chao1, Simpson, and Shannon. ACE and Chao1 are indices for evaluating the number of OTUs contained in the sample. Simpson and Shannon indices were used to reflect the diversity of the sample population: the larger the Simpson, the lower the diversity of the flora, and the larger the Shannon, the higher the diversity of the flora. The Wilcoxon rank sum test found that there was no significant difference between the RS1 group and RS2 group, *P* = 0.2328: Shannon index, *P* = 0.2169; Simpson index, *P* = 0.2017; ACE, *P* = 0.3669; and Chao1, *P* = 0.5125. There was no significant difference in diversity between RS1 and RS2 (Table 2).

**Table 2:** Sequencing results and diversity index of two groups of samples. *The operational taxonomic units (OTUs) were defined at the 97% similarity level

	Group		P value
	RS1	RS2	
ACE	2032.94 ± 1342.18	1468.45 ± 1207.79	0.37
Chao1	2200.16 ± 1437.12	1664.03 ± 1300.57	0.51
Simpson	0.91 ± 0.12	0.86 ± 0.13	0.20
Shannon	6.87 ± 2.73	5.28 ± 2.44	0.22

### Changes in microbial community composition after hysterectomy

The relevant bacterial composition of the patient was analyzed from the perspective of phylogeny (domain/phylum/class/order/family/genus). In the human microbiota, the bacterial group is quite conservative, which can directly reflect the heterogeneity of bacterial community structure in different human body parts. Therefore, when analyzing the composition of common human microorganisms, we must first explain the relative abundance of bacterial phyla. The law of variation, in the bacterial classification unit, the phylum can be called the highest classification unit. Species annotations were made by comparison with the Silva 132 database, and statistics were analyzed at different classification levels: there were 6651 OTUs, of which all could be annotated to the database (100.00%), and the proportion of annotations to the boundary level was 100.00%. The ratio of the phylum level is 91.35%, the ratio of the class level is 81.01%, the ratio of the order level is 69.59%, the proportion of the family level is 58.39%, the proportion of the genus level is 33.82%. According to the results of the species annotation, each species or group is selected in the top 10 species of the highest abundance in the horizontal phylum, class, order, family, genus, and the relative abundance of the species is generated. A cylindrical cumulative graph to visually view the species and their proportions of the relatively abundant abundance of each sample at different classification levels.

### Analysis at the phylum level

**Fig. 2:**
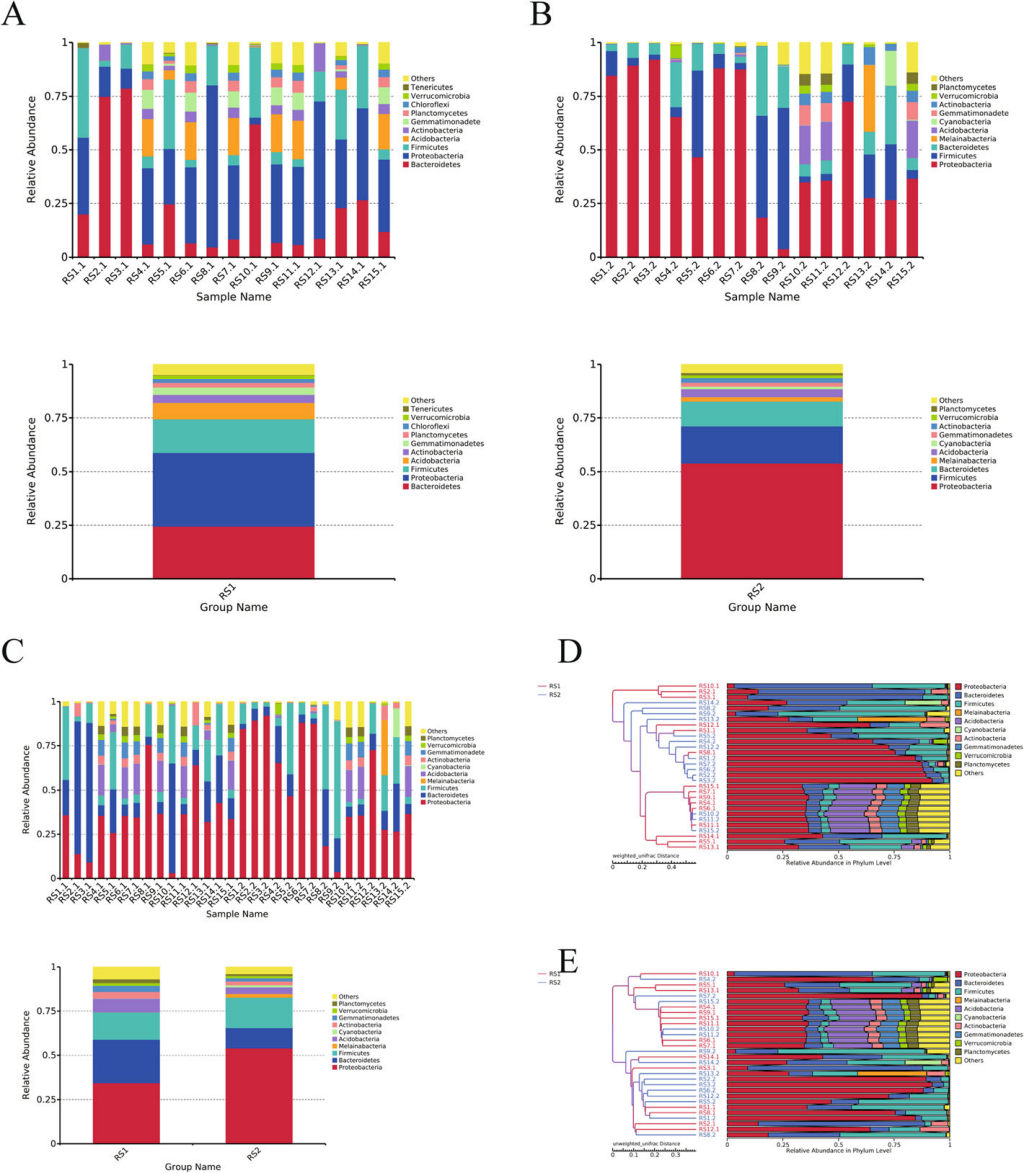
Changes in microbial community composition after hysterectomy at the level of phylum.**a** The top ten strains of the RS1 group ranked as the most abundant.**b** The top ten strains of the RS2 group ranked as the most abundant.**c** At the phylum level, the three dominant bacteria, *Bacteroidetes*, *Proteobacteria* and *Firmicutes*, accounted for more than 75% of the intestinal flora .**d** UPGMA clustering tree based on the Weighted Unifrac distance.**e** UPGMA clustering tree based on the Unweighted UniFrac distance

At the phylum level, the top ten strains of the RS1 and RS2 groups ranked as the most abundant. In the RS1 group, they were: *Bacteroidetes*, *Proteobacteria*, *Firmicutes*, *Acidobacteria*, *Actinobacteria*, *Gemmatimonadetes*, *Planctomycetes*, *Chloroflexi*, *Verrucomicrobia*, *Tenericutes*, and bacteria that could not be classified (Fig. 2A). In the RS2 group, they were: *Bacteroidetes*, *Proteobacteria*, *Firmicutes*, *Melainabacteria*, *Acidobacteria*, *Cyanobacteria*, *Gemmatimonadetes*, *Actinobacteria*, *Verrucomicrobia*, *Planctomycetes*, and bacteria that could not be classified (Fig. 2B). At the phylum level,the dominant bacteria were basically the same, and the three dominant bacteria, *Bacteroidetes*, *Proteobacteria* and *Firmicutes*, accounted for more than 75% of the intestinal flora (Fig. 2C). Significant individual differences occurred between the samples. The proportion of *Bacteroidetes* in each sample ranged from 2.85 to 78.77%, and the proportion of *Proteobacteria* in each sample ranged from 3.15 to 92.13%. *Firmicutes* were present in each sample at a proportion of 2.31 to 66.03%, and the relative abundance of *Bacteroidetes* was higher in the RS1 group than RS2 group, *P* = 0.003. After surgery, *Bacteroidetes* decreased significantly, and the relative abundance of *Proteobacteria* was significantly lower in the RS1 group, *P* = 0.016, and thus increased after surgery. The relative abundance of *Firmicutes* was lower in the RS1 group but not significantly, *P* = 0.926 (Table 3); combined with UPGMA (Unweighted Pair-group Method with Arithmetic Means) clustering tree, in environmental biology, UPGMA is a commonly used clustering analysis method. It is the earliest method used to solve a classification problem. The UPGMA clustering analysis was performed with the Weighted UniFrac distance matrix and the Unweighted UniFrac distance matrix, and the clustering results were integrated with the relative abundance of the species at the phylum level (Fig. 2D, E, suggesting that the structure of the two groups of bacteria is not significantly different.

**Table 3:** Comparison of relative abundance of two groups of TOP3 strains at the phylum level

	Group		P value
	RS1	RS2	
Bacteroidetes(%)	24.54	11.43	0.003
Proteobacteria(%)	34.36	54.04	0.016
Firmicutes(%)	0.003	17.26	0.926

### Analysis at the class level

**Fig. 3:**
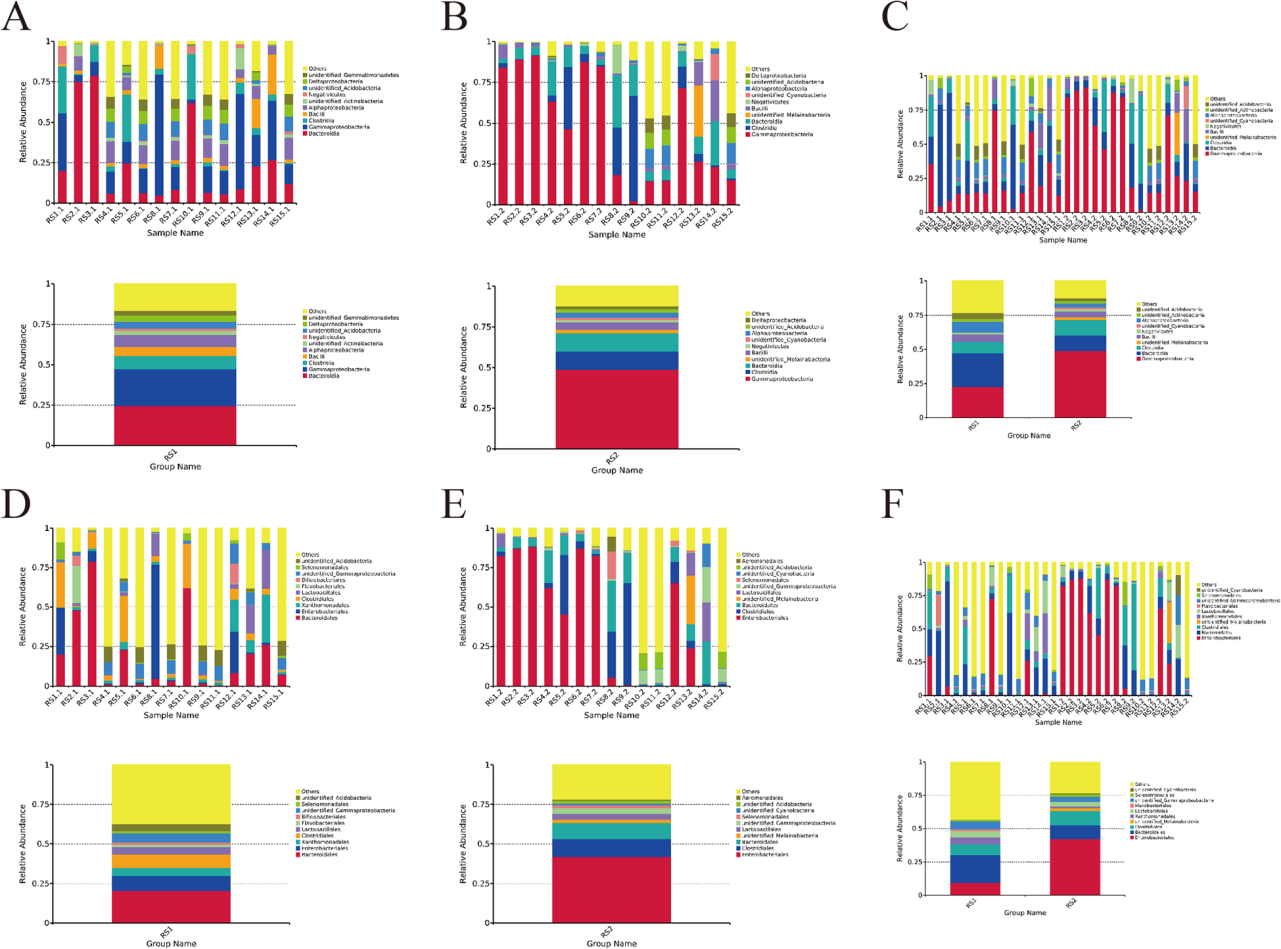
Changes in microbial community composition after hysterectomy at the level of calss and order.**a** The top ten strains of the RS1 group ranked as the most abundant at the level of class.**b** The top ten strains of the RS2 group ranked as the most abundant at the level of class.**c** At the class level, the three dominant bacteria, *Gammaproteobacteria*, *Bacteroidia* and *Clostridia*, accounted for more than 50% of the intestinal flora.**d** The top ten strains of the RS1 group ranked as the most abundant at the level of order.**e** The top ten strains of the RS2 group ranked as the most abundant at the level of order.**f** The dominant species at the order level were *Enterobacteriales*, *Bacteroidales* and *Clostridiales*

At the class level, the top ten strains in the RS1 and RS2 groups were selected. The RS1 group included: *Bacteroidia*, *Gammaproteobacteria*, *Clostridia*, *Bacilli*, *Alphaproteobacteria*, *unidentified_Actinobacteria*, *Negativicutes*, *unidentified_Acidobacteria*, *Deltaproteobacteria*, *unidentified_Gemmatimonadetes*, and bacteria that could not be classified (Fig. 3A). The RS2 group included: *Gammaproteobacteria*, *Clostridia*, *Bacteroidia*, *unidentified_Melainabacteria*, *Bacilli*, *Negativicutes*, *unidentified_Cyanobacteria*, *Alphaproteobacteria*, *unidentified_Acidobacteria*, *Deltaproteobacteria*, and bacteria that could not be classified (Fig. 3B). The dominant species at the class level were *Gammaproteobacteria*, *Bacteroidia* and *Clostridia*, which accounted for more than 50% of the intestinal flora (Fig. 3C). Significant individual differences occurred between the samples. *Gammaproteobacteria* accounted for 2.04–91.43% of each sample, and *Bacteroidia* accounted for 2.85–78.77% of each sample, while *Clostridia* accounted for 0.37–64.80% of each sample.

### Analysis at the order level

At the order level, the top ten strains in the RS1 and RS2 groups were selected. The RS1 group included: *Bacteroidales*, *Enterobacteriales*, *Xanthomonadales*, *Clostridiales*, *Lactobacillales*, *Flavobacteriales*, *Bifidobacteriales*, *unidentified_Gammaproteobacteria*, *Selenomonadales*, *unidentified_Acidobacteria*, and other bacteria that could not be classified (Fig. 3D). The RS2 group included: *Enterobacteriales*, *Clostridiales*, *Bacteroidales*, *unidentified_Melainabacteria*, *Lactobacillales*, *unidentified_Gammaproteobacteria*, *Selenomonadales*, *unidentified_Cyanobacteria*, *unidentified_Acidobacteria*, *Aeromonadales*, and other bacteria that could not be classified (Fig. 3E). The dominant species at the order level were *Enterobacteriales*, *Bacteroidales* and *Clostridiales* (Fig. 3F).

### Analysis of species differences and differences between species

**Fig. 4:**
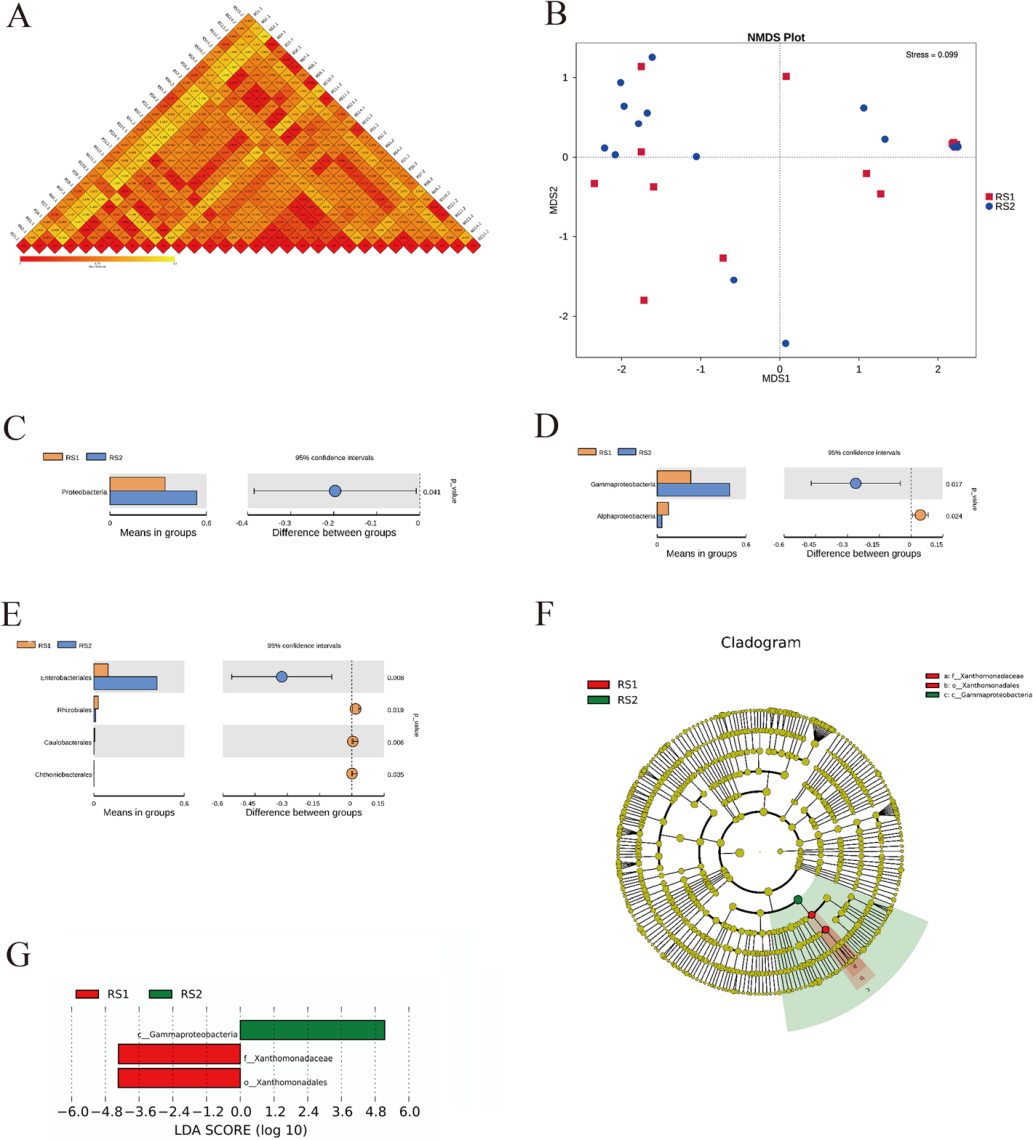
Analysis of species differences and differences between species.**a** Beta diversity heatmap: The numbers in the boxes in the figure are the dissimilarity coefficients between the samples. The disparity coefficients are smaller, the difference in species diversity is smaller. In the same box, the upper and lower values represent Weighted Unifrac and Unweighted Unifrac distance.**b** NMDS:Each point in the figure represents a sample. The distance between the points represents the degree of difference, and the same group of samples is represented by the same color.**c** Analysis of species differences between T_test groups at the phylum level.**d** Analysis of species differences between T_test groups at the class level.**e** Analysis of species differences between T_test groups at the order level.**f** Cladogram:In the cladogram, the circle radiating from the inside to the outside represents the classification level from the phylum to the genus (or species). Species with no significant difference are uniformly colored yellow. Different species of Biomarker follow the group for coloring. The red nodes indicate the microbial groups that play an important role in the red group, and the green nodes indicate the microbial groups that play an important role in the green group.**g** The LDA value distribution histogram

The weighted UniFrac distance and the unweighted UniFrac distance were used to measure the difference coefficient between the two samples (Fig. 4A). The unweighted UniFrac distance was tested by the Wilcoxon rank sum test, *P* = 0.4646, and the weighted UniFrac distance was also tested by the Wilcoxon rank sum test, *P* = 0.1083, indicating that there was no significant difference in species diversity between the two groups.

NMDS (Non-Metric Multi-Dimensional Scaling) statistics is a sorting method suitable for ecological research. The smaller the stress (< 0.2), the more accurately. Stress = 0.156 indicated that NMDS accurately reflected the degree of difference between samples (Fig. 4B).

MRPP (Multi Response Permutation Procedure) analysis was used to analyze whether the differences in microbial community structure between groups were significant. A value of less than 0.05 indicates a significant difference. Table 4 showed that the differences between the two groups were significant.

**Table 4:** Significantness test table for community structure differences between groups

Group	A	Observed-delta	Expected-delta	Significance
RS1-RS2	0.03	0.80	0.82	0.036

To find the differential species between the groups at each classification level (phylum, class, order), a t-test test between the groups was performed to determine the species with significant differences (*P* < 0.05).

At the phylum level, the species difference analysis between the t-test groups was obtained. *Proteobacteria* showed a significant difference between the two groups. The average abundance of the RS1 group was 34.36%, and the average abundance of the RS2 group was 54.04%, *P* < 0.05 (Fig. 4C).

At the class level, the difference in species between the t-test groups was obtained. *Gammaproteobacteria* (*Proteobacteria*) showed a significant difference between the two groups; the average abundance of RS1 was 22.74%, and the average abundance of RS2 was 48.89%, *P* < 0.05. There was a significant difference between the groups for *Alphaproteobacteria* (*Proteobacteria*); the average abundance of RS1 was 7.56%, and the average abundance of RS2 was 3.20%, *P* < 0.05 (Fig. 4D).

At the order level, the difference in species between the t-test groups was determined. For *Enterobacteriales* (*p__Proteobacteria*; *c__Gammaproteobacteria*), the average abundance of RS1 was 9.44%, and the average abundance of RS2 was 42.05%, *P* < 0.05, indicating a significant difference between the two groups. For *Rhizobiales* (*p__Proteobacteria*; *c__Alphaproteobacteria*), the average abundance of RS1 was 2.85%, and the average abundance of RS2 was 1.10%, *P* < 0.05, indicating a significant difference between the two groups. For *Caulobacterales* (*p__Proteobacteria*; *c__Alphaproteobacteria*), the average abundance of RS1 was 0.58%, and the average abundance of RS2 was 0.13%, *P* < 0.05, indicating a significant difference between the two groups. The average abundance of RS1 for *Chthoniobacterales* (*p__Verrucomicrobia*; *c__Verrucomicrobiae*) was 0.13%, and the average abundance of RS2 group was 0.04%, *P* < 0.05, indicating a significant difference between the two groups (Fig. 4E).

LEfSe (LDA (Linear Discriminant Analysis) effect size) is used to compare two or more groups. The LDA value distribution histogram shows the species with an LDA score greater than the set value (the default setting is 4), i.e., the biomarker with statistical differences between the groups. This study showed that the species with significant differences in abundance in the different groups were *c_Gammaproteobacteria*, *f_Xanthomonadaceae*, and *o_Xanthomonadales*, and the length of the histogram bar represents the size of the difference species (i.e., LDA score). *C_Gammaproteobacteria* was enriched in the RS2 group, and *f_Xanthomonadaceae* and *o_Xanthomonadales* were enriched in the RS1 group; the LDA score showed *f_Xanthomonadaceae* > *o_Xanthomonadales*, indicating great influence of *f_Xanthomonadaceae* in the RS1 group (Fig. 4F, G).

## Discussion

High-throughput sequencing technology is a presently widely used sequencing technology, which can quickly and accurately sequence a large number of samples simultaneously, and obtain a large amount of data. High-throughput sequencing is particularly important in the gut flora. This technology has the advantages of high accuracy and high sequencing depth, which can detect low abundance or unknown bacteria, and further obtain more accurate and comprehensive flora information. The intestinal flora is a special system that coexists with the human body. This has a large number and variety, and is an ecosystem with a high density of cells. In recent years, as the research on the intestinal flora become more and more intensive, studies have revealed that the intestinal flora is correlated to a variety of diseases, including intestinal tumors, autism, obesity and diabetes. The intestinal flora can change through the interaction of hormones in the body and in vitro, and this can affect the ecological balance in the body. If the intestinal flora in the body becomes imbalanced, this would cause an adverse effect on intestinal function. The literature indicates that the use of exogenous hormones can cause the imbalance of the intestinal flora, and increase the diversity of the flora. The adult gut flora contains approximately 1000 different bacterial species, in which thick-walled bacteria (such as *Clostridium*, *Enterococcus* and *Lactobacillus*), *Bacteroidetes* (such as *Prevotella* and *Bacteroidetes*) and *Actinobacteria* (such as *Bifidobacteria*) are the major members [22]. In recent years, the determination of whether the intestinal flora can regulate estrogen and its metabolites has attracted the attention of scholars. Glucuronidase in the intestinal flora can promote the reabsorption of estrogen, and the level of estrogen is closely correlated to the occurrence of uterine fibroids [23]. The study [24] conducted by Plottel et al. and other studies have found that a variety of bacteria in the intestinal flora are associated with estrogen metabolism, and all genes in the bacteria that are capable of metabolizing estrogen were collectively referred to as the estrobolome. The high bacterial enzyme activity of the estrobolome causes the free estrogen in the enterohepatic circulation to significantly rise, thereby forming an endogenous hormonal environment. This endogenous hormonal environment significantly increases the risk of hormone-dependent tumors, including breast and endometrial cancers, through direct or indirect effects [25]. The study conducted by Guo et al. revealed the relationship between PCOS (polycystic ovary syndrome) and the intestinal flora. Estrone and E2 levels were lower in the PCOS group than in the normal control group, and this shows that the intestinal flora can affect the occurrence and treatment of PCOS [26].

The present study focused on the changes in the intestinal flora of patients with factor hysteromyoma before and after total hysterectomy. Uterine fibroids are benign tumors in women, who are highly dependent on estrogen and progesterone in the body. Although the uterus does not secrete hormones, the uterus is the main receptor organ of estrogen in the body. The anatomical structure of the uterus and ovary is closely correlated. Except for the ovarian blood supply from the ovarian artery, a considerable part of the blood supply comes from the ascending branch of the uterine artery. The scope of the total hysterectomy includes the uterus and its surrounding ligaments. At the same time, due to some unavoidable thermal damage during the operation, which can damage the surrounding vascular tissues, the patient’s ovarian blood supply is affected after the total hysterectomy, thereby leading to a decrease in sex hormone secretion [27]. The ovary has two functions of ovulation and secretion of hormones. The indicators that respond to ovarian function include E2, FSH and AMH. If ovarian function is reduced, E2 and AMH are reduced, and FSH is increases. The statistics of the present study suggest that the levels of E2 and AMH in patients undergoing abdominal hysterectomy were significantly higher in the preoperative group than in the postoperative group, while the levels of FSH in the preoperative group were lower than those in the postoperative group, and the differences between these two groups were statistically significant. This indicates that hysterectomy damages the function of ovarian secretion to a certain extent, and that the estrogen reduction is more significant. A study [28] also reported that patients with uterine fibroids have a greater effect on ovarian function after hysterectomy, when compared to patients with uterine fibroids removal.

Some literatures have suggested that the richness of the intestinal flora is closely correlated to systemic estrogen. The richness of the flora at the phylum level does not affect the content of estrogen and estrogen metabolites in the body. However, a large number of bacteria at the family and species level regulate the content of estrogen. In particular, *Clostridium* and *Pneumococcus* have the most significant effect on estrogen metabolism [29]. In recent years, there have been many studies on the intestinal flora and various systemic diseases, but there are few literatures on the changes of the intestinal flora after total hysterectomy. According to the results of the present study, the flora coverage of these two groups of samples reached more than 98%, indicating that the flora coverage was good. From the perspective of the diversity of the flora, the analysis of the alpha diversity analysis index (Shannon index, Simpson index, ACE index and Chao1 index) indicated that there was no statistically significant difference between the RS1 group and RS2 group. This shows that there were no significant differences between these two groups of microbiome alpha diversity. Although the estrogen level decreased and the ovarian function was reduced after the total hysterectomy, the diversity of the intestinal flora before and after surgery was less different for patients.

For these patients, the level of estrogen in the body decreased after the total hysterectomy. In order to further explore the predominant strains of these two groups before and after surgery, further exploration was conducted in the present study. Based on the composition of the flora, the dominant strains in the RS1 and RS2 groups were identified. At the phylum level, the top three dominant strains in the RS1 and RS2 groups were *Bacteroidetes*, *Proteobacteria* and *Firmicutes*. The abundance of *Bacteroidetes* RS1 was significantly higher than that of RS2, and the abundance of *Proteobacteria* RS1 was significantly lower than that of RS2. *Gammaproteobacteria*, *Bacteroidia* and *Clostridia* dominated at the level of the class. *Enterobacteriales*, *Bacteroidales* and *Clostridiales* were the dominant species at the order level. At the phylum level, the species with differences between the RS1 and RS2 groups was *Proteobacteria*. At the class level, the species with statistically significant differences between these two groups were *Gammaproteobacteria* (*Proteobacteria*) and *Alphaproteobacteria* (*Proteobacteria*). At the level of the order, the species with statistically significant differences between these two groups were *Enterobacteriales* (*p__Proteobacteria*, *c__Gammaproteobacteria*), *Rhizobiales* (*p__Proteobacteria*, *c__Alphaproteobacteria*) and *Caulobacterales* (*p__Proteobacteria*, *c__Alphaproteobacteria*). Some studies have revealed that a decrease in estrogen level leads to a decrease in the diversity of the intestinal flora and a reduction in the abundance of thick-walled bacteria, including *Clostridium* [30]. The decrease in estrogen levels in the present study lead to the increase in abundance of *Firmicutes*, the decrease in diversity of *Bacteroidetes*, and the increase in species diversity of *Proteobacteria*. However, a study conducted in 2014 [31] revealed that the abundance of estrogen and its metabolites, and the intestinal flora in the phyla, class and genus categories were correlated. Furthermore, it was noted that *Clostridiales* and *Ruminococcaceae* under *Firmicutes* are positively correlated with estrogen metabolites, but negatively correlated with *Bacteroidetes*. In general, for patients who received total hysterectomy, the composition of the intestinal flora changes with the increase in *Proteobacteria*.

The MRPP analysis revealed that the differences between the RS1 and RS2 groups were greater than the intra-group differences, and that the differences between these groups were statistically significant. Based on the Unifrac distance for PoCA (Principal Co-ordinates Analysis) analysis, the PC1 factor expressed in 39.2%, and the NMDS could accurately reflect the degree of difference between these two groups of samples. At the same time, the level of estrogen in the body decreased after the total hysterectomy. It was assumed that the total hysterectomy was the cause of the intestinal flora.

The level of estrogen in the body can change the intestinal flora, but the manner in which the intestinal flora regulates estrogen metabolism in the body remains unclear. Therefore, further research is needed. Although the present study proposed the decrease in estrogen level and a series of changes in the intestinal flora after the total hysterectomy, there were still limitations in the present study that needs further exploration. The present study lacks a comparative analysis of sex hormones and the intestinal flora in patients with uterine fibroids and healthy women. At the same time, due to the small sample size, there may be some bias in the experimental results. In the future, the sample size needs to be expanded for a more in-depth research.

## Conclusion

In conclusion, transabdominal hysterectomy can reduce estrogen levels in the body, and reduce the diversity and abundance of the intestinal flora before and after surgery, but the main difference was the increase in *Proteobacteria*. In the future, more multi-center and large-sample studies are needed to obtain more accurate conclusions, and further investigate the interaction mechanism between the intestinal flora and estrogen. A more rigorous and reliable scientific basis for patients with factor hysteromyoma, undergoing total hysterectomy after application of hormone replacement, when necessary, as well as dietary guidance, should be provided.

## Methods

### Patient enrollment and sample collection

This was a case-control study that included women aged 40–45 years who underwent transabdominal hysterectomy due to uterine fibroids from September 2018 to December 2018 in the Department of Obstetrics and Gynecology, Shengjing Hospital of China Medical University. Inclusion criteria were: patients with uterine fibroids with ultrasound and clinical diagnosis; no hypertension, diabetes, heart disease or other comorbidities; no menopause; no previous abdominal surgery or intestinal disease history; consistent intensity of antibiotic use after surgery; and no significant change in bowel habits after surgery. The patients were divided into the preoperative RS1 group, and the postoperative RS2 group. Blood for E2, AMH and FSH tests and stool specimens were collected from the RS1 and RS2 groups. This study was approved by the Ethics Committee of Shengjing Hospital of China Medical University.

### Sample collection, DNA extraction and 16S rRNA gene amplicon sequencing

Fresh stools were collected from 15 patients before and at one month after the surgery. Approximately 5 g of the middle part of the stool was placed in a sterile dry test tube containing pure ethanol, and stored in a freezer at − 80 °C. The CTAB (Cetyltrimethylammonium Ammonium Bromide) or SDS (Sodium dodecyl sulfate) method was used to extract the genomic DNA of the sample, and the purity and concentration of the DNA were detected by agarose gel electrophoresis. Afterwards, an appropriate amount of the sample was collected in a centrifuge tube, and the sample was diluted to 1 ng/μl with sterile water. Then, these samples were amplified by PCR (polymerase chain reaction). Next, equal concentration mixing was performed, according to the PCR product concentration. After thorough mixing, 2% agarose gel electrophoresis was used to purify the PCR products. The sequence with a main band size within 400–450 bp was select, and this was tapped to recover the target band. The library was constructed using the NEB Next® Ultra™ DNA Library Prep Kit for Illumina (New England Biolabs). The constructed library was subjected to Qubit quantification and library detection. After the qualification, HiSeq was used for the on-line sequencing [32, 33]. Blood samples were taken on an empty stomach at one day before the surgery, and at one month after the surgery, in order to detect the E2, AMH and FSH. E2 and FSH were detected using the Beckman Coulter UniCel DXI 800. The detection method used was chemiluminescence. AMH was detected by ELISA (enzyme-linked immunosorbent assay).

### Analysis of 16S rRNA gene sequences, bioinformatics and statistical analyses

The sequences were analyzed using the QIIME [34] (Quantitative Insights Into Microbial Ecology) software package, and in-house Perl scripts were used to analyze the alpha-diversity (within samples) and beta-diversity (among samples). First, the reads were filtered using QIIME quality filters. Then, pick_de_novo_otus.py was use to select the OTUs by making an OTU table. Sequences with ≥97% similarity were assigned to the same OTUs. A representative sequence was selected for each OTU, and the RDP classifier [35] was used to annotate the taxonomic information for each representative sequence. OTUs that reached a 97-nucleotide similarity level were used for the alpha diversity (Shannon, Simpson index) and richness analysis (ACE and Chao1). Then, rarefaction curves were generated based on these three matrices. A metagenomic biomarker discovery approach was employed with LEfSe (linear discriminant analysis [LDA] coupled with effect size measurement), which performed with the nonparametric Wilcoxon sum-rank test. In order to mine deeper data of the microbial diversity of the differences between these samples, a significance test were conducted with some statistical analysis methods, including *t*-test, LEfSe, ANOSIM and MRPP. Beta diversity was used to explore the differences between samples, and the Wilcoxon rank sum test was used to determine whether the differences in beta diversity between these groups were significant. The SPSS 24.0 software was used for data processing. The measurement data were expressed as mean ± standard deviation. *T*-test was used for comparisons between two groups. The count data was used to indicate the ratio. Chi-square test was used for comparisons between two groups. *P* < 0.05 was considered statistically significant.

## Data Availability

The datasets generated and analyzed are available from corresponding author on reasonable request.

## Abbreviations

TNF-α: Tumor Necrosis Factor-α
EGF: epidermal growth factor
VGEF: vascular endothelial growth factor
TGF: transforming growth factor
E2: estradiol
AMH: anti-Mullerian hormone
FSH: follicle-stimulating hormone
OTUs: Operational Taxonomic Units
UPGMA: Unweighted Pair-group Method with Arithmetic Means
NMDS: Non-Metric Multi-Dimensional Scaling
MRPP: Multi Response Permutation Procedure
LDA: Linear Discriminant Analysis
LEfSe: LDA effect size
PCOS: polycystic ovary syndrome
PoCA: Principal Co-ordinates Analysis
PCR: polymerase chain reaction
ELISA: enzyme-linked immunosorbent assay
CTAB: Cetyltrimethylammonium Ammonium Bromide
SDS: Sodium dodecyl sulfate
QIIME: Quantitative Insights Into Microbial Ecology
